# Patients' opinion on the barriers to diabetes control in areas of conflicts: The Iraqi example

**DOI:** 10.1186/1752-1505-2-7

**Published:** 2008-06-24

**Authors:** Abbas Ali Mansour

**Affiliations:** 1 Assistant Professor of Medicine, Department of Medicine, Basrah College of Medicine, Basrah, Hattin post office P.O Box: 142 Basrah, 42002, Iraq

## Abstract

**Background:**

The health system in Iraq has undergone progressive decline since the embargo that followed the second gulf war in 1991. The aim of this study is to see barriers to glycemic control form the patient perspective, in a diabetic clinic in the south of Iraq.

**Methods:**

A cross sectional study from the diabetes out-patient clinic in Al-Faiha general hospital in Basrah, South Iraq for the period from January to December 2007. The study includes diabetic patients whether type 1 or 2 if they have at least one year of follow up in the same clinic. Those with A1C ≥ 7% were interviewed by special questionnaire, that was filled in by the medical staff of the clinic. The subjects analyzed in this study were adults (≥ 18 years old) with previously diagnosed diabetes (n = 3522). The duration of diabetes range from 1 to 30 years.

**Results:**

Mean A1C was 8.4 ± 2 percent, with 835(23.7%) patients with A1C less than 7% and 2688(76.3%) equal to or more than 7%. Of 3522 studied patients, 46.6% were men and 51.5% were women, with mean age of 53.78 ± 12.81 year and age range 18–97 years. Patient opinion for not achieving good glycemic control among 2688 patients with HbA1C ≥ 7% included the following. No drug supply from primary health care center (PHC) or drug shortage is a cause in 50.8% of cases, while drugs and or laboratory expense were the cause in 50.2%. Thirty point seven percent of patients said that they were unaware of diabetics complications and 20.9% think that diabetes is an untreatable disease. Thirty percent think that non-control of their diabetes is due to migration after the war. No electricity or erratic electricity, self-monitoring of blood glucose (SMBG) is not available, or strips were not available or could not be used, and illiteracy as a cause was seen in 15%, 10.8% and 9.9% respectively.

**Conclusion:**

Our patients with diabetes mellitus declared that of the causes for poor glycemic control most of them related to the current health situation in Iraq.

## Background

The health system in Iraq underwent progressive decline since the embargo that followed the second gulf war in 1991. The war in 2003, exacerbated that by causing further damage to the infrastructure, with lack of security that making even drug distribution unsafe, with further deterioration due to electricity problems [1-3]. This makes drug storage even more difficult.

Reports by the United Nations assistance mission for Iraq indicate that the war in Iraq caused hundreds of thousands of civilians have been displaced, and that military operations in the country are limiting civilian access to health and education services, food, electricity and water supplies [3]. Currently, the Iraqi health system is unable to cope with the health care needs of its population [2,4].

Attaining glycemic control (defined as a A1C concentration of less than 7.0%) is imperative for the delay or prevention of diabetes related complications, which are the real dangers of type 2 diabetes [5,6].

For each 1% reduction in the mean A1C, there was a 21% risk reduction for any diabetes-related end point, including myocardial infarction, stroke, amputation, and microvascular complications [7].

Despite the increasing prevalence of diabetes, improved understanding of the disease, and a variety of new medications, glycemic control does not appear to be improving even in developed nations [8].

Most diabetic patients are likely to encounter barriers to care that pose major challenges in adhering to self-management programmes[9]. Determining the barriers to achieving optimal glycemic control is important in enabling patients to do better in terms of improving diabetes control and thereby reducing risk of longer-term complications[10]. The most frequently reported barriers are time constraints, knowledge deficits, limited social support, inadequate resources, limited coping skills, poor patient-provider relationship and low self-efficacy[11,12].

General practitioners (GPs) often assume that the best methods to increase compliance/adherence are shocking the patients, putting pressure on them and threatening to refer them to hospital in a study of GPs' perspectives of type 2 diabetes patients' adherence to treatment[13]. The problems and barriers perceived by GPs providing diabetes care in primary care in England and Wales were lack of time/under-funding and keeping up to date in the area of diabetes, followed by lack of space, inadequate chiropody, dietetics, ophthalmology and access to secondary care[14].

Of a population of 27 million Iraqi populations, the prevalence of type 2 diabetes is reaching epidemic proportions, impacting an estimated 2 million people–7.43% of the overall Iraqi population[15].

The aim of this study is to see barriers to glycemic control form the patient perspective in a diabetic clinic in the south of Iraq.

## Methods

Participants were recruited in this cross-sectional study from the diabetes out-patient clinic in Al-Faiha general hospital in Basrah, Southern Iraq for the period from January to December 2007.

The study includes diabetic patients whether type 1 or 2 if they had at least one year of follow up in the same clinic. Those with A1C ≥ 7% were interviewed by special questionnaire that was filled out by the medical staff of the clinic. Overall, 8 questions were present in the questionnaire. Patients were asked to mention the main causes of poor glycemic control from these 8 questions, and to choose more than one answer according to their wishes. The answers were yes or no. These questionnaires where suggested from the patients opinion for the cause of poor glycemic control of the last year preceding this study.

All the patients agreed to participate in the study with written informed consent taken. Ethical approval was taken from the local ethical committee in Basrah directorate of health.

Exclusion criteria were age less than 18 years, pregnant women, and patients with a history of diabetes for less than 1 year, less than one year of follow up in the clinic or those had no value of A1C.

The subjects analyzed in this study were adults (≥ 18 years old) with previously diagnosed diabetes (n = 3522). The duration of diabetes ranged from 1 to 30 years.

Lifestyle modification where used for of our patients with oral antidiabetic drugs (OAD), metformin unless there was high serum creatinine levels ≥ 132.6 μmol/L (1.5 mg/dl) according to guidelines [16].

Smokers were considered for any one who had smoked at least 1 cigarette in the past 3 months.

### Anthropometric measurements

Waist circumference (WC) was measured at the umbilical level from the horizontal plane in centimeters (cm), using a plastic anthropometric tape with the subjects standing and breathing normally by the same physician during the physical examination with the participant standing erect. Standing height and weight measurements were completed with the subjects wearing lightweight clothing and no shoes. Height was measured to the nearest cm and weight was measured to the nearest half kilogram (kg). Body mass index (BMI) was calculated as body weight in kilograms divided by the squared value of body height in meters (kg/m2). Waist to hip ratio (WHpR) and waist to height ration (WHtR) were measured accordingly as ratios.

Blood pressure was measured with a mercury sphygmomanometer on the right arm with the subjects in a sitting position after a 5 min rest. Hypertension was defined as systolic blood pressure ≥ 140 mmHg and/or diastolic blood pressure ≥ 90 mmHg and/or current medication with antihypertensive drugs.

Coronary heart disease diagnosis was based on a history of admission to CCU with elevated cardiac biomarkers, electrocariographic evidence of Q wave myocardial infarction or left bundle branch block, echocardiographic segmental wall motion abnormalities, abnormal angiocardiography, percutaneous coronary intervention or coronary artery bypass surgery. Cerebrovascular disease was diagnosed on the basis of sudden neurologic deficit that lasted for 24 hours with or without neuroimaging changes. Proteinuria was considered on the basis of persistent frank proteinuria without RBC or WBC in urine.

All measurements of A1C were performed in a laboratory using an ion-exchange HPLC method, whose upper reference limit was 5.8%.

### Statistical analysis

Patients' characteristics were reported as percentages or mean ± standard deviation. Statistical analysis was performed using SPSS for WINDOWS (SPSS Inc., Chicago, IL, USA). Two-sample comparisons of individual characteristics were performed by Student's t-test or x2 test. Differences were considered significant at the P < 0.05 level for all these tests.

Patients' characteristics were reported as percentages or mean ± standard deviation.

## Results

Mean A1C was 8.4 ± 2 percent, with 835 (23.7%) patients having A1C less than 7% and 2688(76.3%) were equal to or more than 7%. Table 1, shows basic study characteristics. Of 3522 studied patients, 46.6% were men and 51.5% were women, with mean age of 53.78 ± 12.81 years and age range 18–97 years. Smokers constituted 20.6% of the study sample. The mean qualification (years of school achievement) was 5.08 ± 5.67 years and 1725(49.0%) were Illiterate. Urban dwellers constituted 60.8%. Mean weight, waist, and BMI were 76.04 ± 16.94 kg, 98.4 ± 12.9 cm and 27.6 ± 5.6 respectively. The WHpR and WHtR were 0.94 ± .07 and 0.59 ± .08 respectively. Type 1 diabetes mellitus constituted for 3.6% and the others were type 2 diabetes mellitus. Insulin with or without OAD was used in 20.8%. Hypertensive constituted 32.1% of the study sample. Coronary heart disease, cerebrovascular disease and proteinuria were seen in 7.2%, 4.3% and 5.3% respectively.

**Table 1:** Baseline study characteristics (n = 3522, aged 18–97 years).

Variables		HbA1C < 7% n = 835(%)	HbA1C ≥ 7 n = 2688 (%)	Total No (%)	P value
Gender	Men	383 (22.8)	1299 (77.2)	1676(47.6)	0.282
	Women	442 (24.3)	1374 (75.7)	1816 (51.5)	
Age		55.14 ± 12.96	53.35 ± 12.73	53.78 ± 12.81	0.622
Smoker		141 (19.4)	585 (80.6)	726(20.6)	0.002
Qualification		5.31 ± 5.80	5.01 ± 5.63	5.08 ± 5.67	0.401
Address	Urban	518 (24.2)	1624 (75.8)	2142(60.8)	0.408
	Rural	317 (23.0)	1063 (77.0)	1380(39.2)	
Weight -kg-(mean ± SD)		76.84 ± 16.32	75.79 ± 17.12	76.04 ± 16.94	0.122
Waist -cm-(mean ± SD)		98.96 ± 12.4	98.3 ± 13.0	98.4 ± 12.9	0.371
BMI		28.09 ± 5.55	27.53 ± 5.62	27.6 ± 5.6	0.988
Waist-hip ratio (mean ± SD)		0.94 ± 0.06	0.94 ± 0.07	0.94 ± .07	0.030
Waist-to-height ratio (mean ± SD)		0.59 ± 0.07	0.59 ± .08	0.59 ± .08	0.903
Type of diabetes	Type 1 diabetes	11 (8.7)	116 (91.3)	127(3.6)	< 0.0001
	Type 2 diabetes	824 (24.3)	2571(75.7)	3395(96.4)	
Therapy	Oral *	744 (26.7)	2044 (73.3)	2788 (79.2)	< 0.0001
	Insulin ± oral	91 (12.4)	643 (87.6)	734(20.8)	< 0.0001
Hypertension		277 (24.5)	855 (75.5)	1132(32.1)	0.471
Coronary heart disease		55 (21.7)	198 (78.3)	253 (7.2)	0.490
Cerebrovascular disease		44 (29.3)	106 (70.7)	150(4.3)	0.116
Proteinuria		48 (25.7)	139 (74.3)	187(5.3)	0.536

* Oral including metformin was used for all except in few with high creatinine or type 1 diabetes.

Table 2, shows patient opinion for not achieving good glycemic control among the 2688 patients with A1C ≥ 7%. No drug supply from primary health care center (PHC) or drug shortage is a cause in 50.8%, while drugs and or laboratory expense were the cause in 50.2%. Thirty point seven percent of patients said that they were unaware of diabetic complications and 20.9% thought that diabetes is an untreatable disease. Thirty percent think that non-control of their diabetes is due to migration after the war. No electricity or erratic electricity, self-monitoring of blood glucose (SMBG) is not available, or no strips were available or could not be used, and illiteracy as a cause was seen in 15%, 10.8% and 9.9% respectively.

**Table 2:** Why do you think that it is difficult to control your diabetes?* (Among 2688 patients with A1C ≥ 7, aged 18–97 years)

Answers	No (%)
1-Illiteracy	268(9.9)
2-No electricity or erratic	403(15)
3-Migration	806(30)
4-Needle phobia	354(13.2)
4-No drug supply from PHC **,or shortage	1365(50.8)
5-Drugs and or laboratory expense	1349(50.2)
6-Unawareness of diabetic complications	825(30.7)
7-Diabetes is untreatable	561(20.9)
8-Self-monitoring of blood glucose (SMBG) is not available, or no strips were available or could not be used.	290(10.8)

*Some have more than one answer.

** PHC -primary health care center

## Discussion

Our diabetic patients are far from achieving glycemic goal since their mean A1C% was 8.4 ± 2, and only 23.7% achieve target glycemic control according to guidelines[5,6]. From the National Health and Nutrition Examination Survey, < 50% of patients with self reported diabetes were at target A1C[17].

Insulin was under used by our patients, only used in 20.8%. In United Kingdom Prospective Diabetes Study over 6 years, ~53% of patients will require addition of insulin therapy to achieve target HbA1C[18].

In Iraq, diabetic patients received their medications including insulin from the PHC that distributed all over, but after the war in 2003, there was catastrophic shortage of drug supply [1]. That's why most patients blame the PHC as a cause of uncontrolled of diabetes. So they buy it from the market, in that case its expensive. Furthermore, people do not always trust governmental hospitals in investigations and they rely on private laboratories which are expensive and that why 50.2% of them blame the expense.

Unawareness of diabetic's complications is a problem in 30.7% and 20.9% thought diabetes is an untreatable disease. Not understanding the nature and consequences of diabetes, as well as a lack of family support, correlated with poor adherence in adults with diabetes[19]. In diabetes care, patients' beliefs about the nature of their illness influence their willingness to adhere to therapy[20]. Unfortunately, there are usually no immediate physical benefits to the treatment of diabetes. Patients who take their diabetes seriously are more likely to adhere to treatment [21]. We have noticed that again as in previous study in Basrah were more than 50% of our patients stopped metformin after a while and more than 80% of those who stopped it, did that with no medical advice to stop it [22].

Migration was blamed in 30% of our study sample. There is more than one type of migration in Basrah after the war, One type is migration from other governorates in Iraq to Basrah and another one is migration within the city. The 3rd type is out side Iraq or to other parts of the country, and we have no data on those because they left.

Needle phobia was a problem in 13.2%. This was problem among 34.7% of 1,267 diabetic patients, in California [23].

Erratic electricity supply no availability of SMBG with illiteracy are problem sizable percents of our study. All guidelines for diabetes management–support the integral role of SMBG in overall treatment programs [5,6].

## Conclusion

Our patients with diabetes mellitus declared that of the causes for poor glycemic control most of them related to the current health situation in Iraq.

## Competing interests

The author declares that they have no competing interests.
